# Academic burnout and coping strategies in healthcare students: a scoping review

**DOI:** 10.1080/10872981.2025.2579392

**Published:** 2025-11-09

**Authors:** Karolina Biaigo, Swagat Ray, Syed Imran Ahmed

**Affiliations:** a School of Health and Care Sciences, College of Health & Science, University of Lincoln, Lincolnshire, United Kingdom; b School of Natural Sciences, College of Health & Science, University of Lincoln, Lincolnshire, United Kingdom

**Keywords:** Academic burnout, coping strategies, healthcare students, scoping review

## Abstract

Burnout is a growing concern among healthcare professionals and students, as it increases the risks of dropping out of higher education, clinical errors and could also lead to maladaptive behaviours such as alcohol abuse. This scoping review aimed to identify all the available literature on effective interventions to address burnout in healthcare students. This scoping review was conducted according to the six-stage protocol by Arksey and O’Malley. Electronic database searches on several platforms, including PubMed/Medline, EMBASE, CINAHL, ERIC, PsycINFO, and Cochrane, for studies published after 2000. Studies were only included if they evaluated the effectiveness of an intervention in addressing burnout among healthcare students using a validated burnout measurement tool, both before and after the intervention. Analysis was carried out using the thematic analysis approach. Searches identified 3517 studies, of which 10 met the inclusion criteria. Three studies were randomised controlled trials, two were comparative cohort studies with an interventional and a control group, and in five studies, one cohort was assessed. Training in mindfulness, emotional intelligence and relaxation techniques significantly reduced burnout. Several interventions, including training in mindfulness and emotional intelligence, can be successfully implemented to reduce burnout in healthcare students. Long-term results are necessary to evaluate the sustainability of benefits associated with these interventions, and moreover, longitudinal studies must demonstrate advantages in dropout rates, employment status, and satisfaction with employment to establish long-term effectiveness in addressing burnout.

## Background

Burnout is defined as an occupational phenomenon characterized by three dimensions: exhaustion (EE), cynicism, also called depersonalization (DP), and a sense of reduced personal accomplishment (PA) [1]. It was first recognized in 1974 by psychologist Herbert Freudenberger, who coined the term due to the severe stress and high standards in healthcare professions, where helping others and self-sacrifice are considered integral parts of the job [2]. While personality traits like ‘the dedicated and the committed’ make people more susceptible [2], burnout remains a complex behavioral syndrome consisting of maladaptive individual responses caused by prolonged exposure to chronic stress [3]. Therefore, changes such as reducing exposure or altering the individual’s response can prevent burnout and lead to healthier outcomes.

Today, burnout is a recognized occupational syndrome, evidenced by the fact that the World Health Organization (WHO) has added burnout to its International Classification of Diseases 11th edition (ICD−11). WHO distinguishes the burnout syndrome from other commonly related mental health disorders, such as stress and depression [1].

Increasingly, burnout affects healthcare professionals early in their careers, including those in training. Bullock et al. demonstrated that healthcare students were more susceptible to stress than their age-matched peers studying non-healthcare degrees [4]. The maladaptive burnout response negatively impacts academic achievement and may even hinder students from qualifying. Therefore, identifying and implementing interventions to reduce burnout early in healthcare careers will build resilience and enhance academic performance and patient care in the future.

When evaluating the impact, burnout negatively affects memory and learning, leading to poor academic performance [5]. In the healthcare education context, burnout is also seen to be associated with academic responsibility [6], unprofessional conduct [7], and even substance misuse [8]. Furthermore, once qualified, healthcare professionals suffering from burnout, particularly the cynicism domain, continue to demonstrate a detached attitude to one’s work, lack in empathy and tolerant patient-centered approach, resulting in poor patient satisfaction and non-adherence to treatment [9,10]. If it is not alarming enough, burnout may also lead to an increased likelihood of clinical errors [11], resulting in compromised patient safety.

Burnout can manifest physically with memory impairment [12], endocrine dysfunction [13] and altered cognition that is demonstrable on R-fMRI [14]. Accompanying these physical symptoms is a maladaptive emotional response. Risk factors for burnout can be defined as intrinsic and extrinsic, and while both categories can be modifiable or non-modifiable, the level of emotional intelligence is seen as a strong modifiable risk factor negatively associated with burnout [15–17]. In a controlled trial by Karahan et al, an 18-hour emotional intelligence training course decreased burnout by 50% [18]. Moreover, emotional intelligence skills increase the likelihood of successful completion of healthcare education [19]. It would therefore be effective and probably cost-effective for this to be delivered as part of the curriculum to mitigate against the negative impact of burnout.

Given the manifestations and impact of burnout, coping strategies are essential. These can be delivered at individual, group and organizational levels, and entail behavioral and cognitive efforts that aim to prevent or treat academic burnout. While evidence suggests, study groups and social support, exercise and other recreational activities, mindfulness and religious rites, alongside professional support such as psychological therapy, are seen as common coping strategies utilized by students [20]. However, there is also an increase in unhealthy strategies, like smoking, excessive eating, alcohol consumption, and gaming [20]. Likewise, at an organizational level, mentorship programs, student wellbeing and counseling services are available through universities [21].

In view of the above, the current review is intended to explore the contemporary literature on burnout in healthcare students and effective methods of tackling it, and to identify gaps requiring future research.

## Methods

This scoping review was approved by the ethics board of the University of Lincoln (2022_10581), and is based on the methodological framework outlined by Arksey and O’Malley, and the additional recommendations by Levac et al on the conduct of a scoping review [22]. Stages outlined in this framework are identifying the research question, identifying relevant studies, study selection, charting the data, collating, summarizing and reporting the results.

### Identifying the research question

The overarching purpose of this review was to map the current literature on effective coping strategies for burnout among healthcare students and to identify gaps requiring future research. To address these goals, the following question was developed: What evidence is available on effective methods of tackling burnout in healthcare students?

### Identifying relevant studies

The purpose of scoping the field is to be as comprehensive as possible in identifying primary studies suitable for answering the central research question. To achieve this, a strategy was adopted that involved identifying eligibility criteria, searching for studies on electronic databases, reviewing the results for eligibility, followed by reviewing the bibliographies of the eligible studies.

#### Eligibility criteria

All English language studies published after the year 2000 AD that examined the effectiveness of any coping strategy for burnout in healthcare students using validated tools for burnout measurement were eligible for inclusion. Examples of validated burnout measurement tools include the Maslach Burnout Inventory (MBI), encompassing 3 scales: emotional exhaustion (EE), depersonalization (DP), and personal accomplishment (PA), as defined by ICD−11 (World Health Organization, 2023). High scores for EE and DP and a low score for PA dimensions of the MBI are indicative of burnout.

The review period after 2000 was selected because there has been a notable rise in mental health disorders over the past two decades [23]. Additionally, the review also considers the impact of the recent COVID−19 pandemic, which has been devastating. To further illustrate, this period also saw significant policy changes in the UK, with increased recognition and support for mental healthcare. In the 2000 NHS Plan, mental health was identified as one of three clinical priorities alongside cancer and heart disease [24,25]. In this plan, the Department of Health announced an extra annual investment of over £ 300 million by the year 2003/04 to ‘fast-forward’ the National Service Framework for Mental Health [25,26].

#### Electronic database search

Electronic database searches were performed on databases, including Medline/PubMed, CINAHL, Embase, PsycInfo, ERIC and Cochrane. The databases were selected to be comprehensive and to cover a broad range of disciplines. The keywords searched using a Boolean search query were: ‘burnout’ AND ‘coping’ OR ‘cope’ OR ‘help’ OR ‘manage*’ AND ‘student’ OR ‘academic’. For each database search, the first step was to search for all titles and abstracts containing the word ‘burnout’. Next, a Medical Subject Headings (MeSH) search of the word ‘student’ in titles and abstracts was performed. MeSH search allows for the simultaneous searching of synonymous words such as pupil, student, academic, and trainee. Next, MeSH searches were performed for the term ‘coping’, and then the results of each search step were combined to generate the final search results.

### Study selection

The search results retrieved were screened for eligibility using a three-stage process. The first stage involved automated electronic deduplication of results. Then, titles and abstracts of the remaining search results were reviewed to eliminate irrelevant material. The full manuscripts of the remaining studies were then reviewed, with those meeting eligibility criteria being selected for inclusion. Finally, the bibliographies of the identified included studies were reviewed for potential missed articles.

### Charting the data

For scoping reviews, data extraction is referred to as ‘charting the data’. The ‘descriptive–analytic’ model described by [27] was utilized for charting the data. This involved charting key terms of information obtained from the primary studies being reviewed. The aim was to chart a summary of the results, which addressed the scoping review's objectives and ideally answered the questions of the review. A charting table was developed using Microsoft Word to identify the key data to collect for each study. In line with best practice guidelines [27], the charted information about each study included:

- Author(s), year of publication
- Title
- Aims
- Study population and sample size
- Methodology
- Intervention type/coping strategy tested
- Key findings

### Collation, summarization and results reporting

This final stage of this study involved collating, summarizing, and reporting the results. Scoping reviews do not seek to ‘synthesize’ the evidence [27], so no attempt was made to present a viewpoint on the weight of evidence in regards to coping strategies for burnout in healthcare students. Furthermore, the review did not seek to assess the quality of evidence; therefore, this study does not determine whether the reviewed studies provide robust or generalizable findings.

A narrative review of the methods to tackle burnout in healthcare students was reported by grouping similar interventions and descriptively summarizing the interventions that were effective in reducing burnout. Because the overall purpose of this scoping review was to explore the breadth of what is currently known about coping strategies for burnout in healthcare students, quality assessment of individual studies was not conducted. Finally, the scoping review was concluded by determining knowledge gaps and discussing areas for future research.

## Results

### Identification of characteristics of potential studies

**Figure 1.**
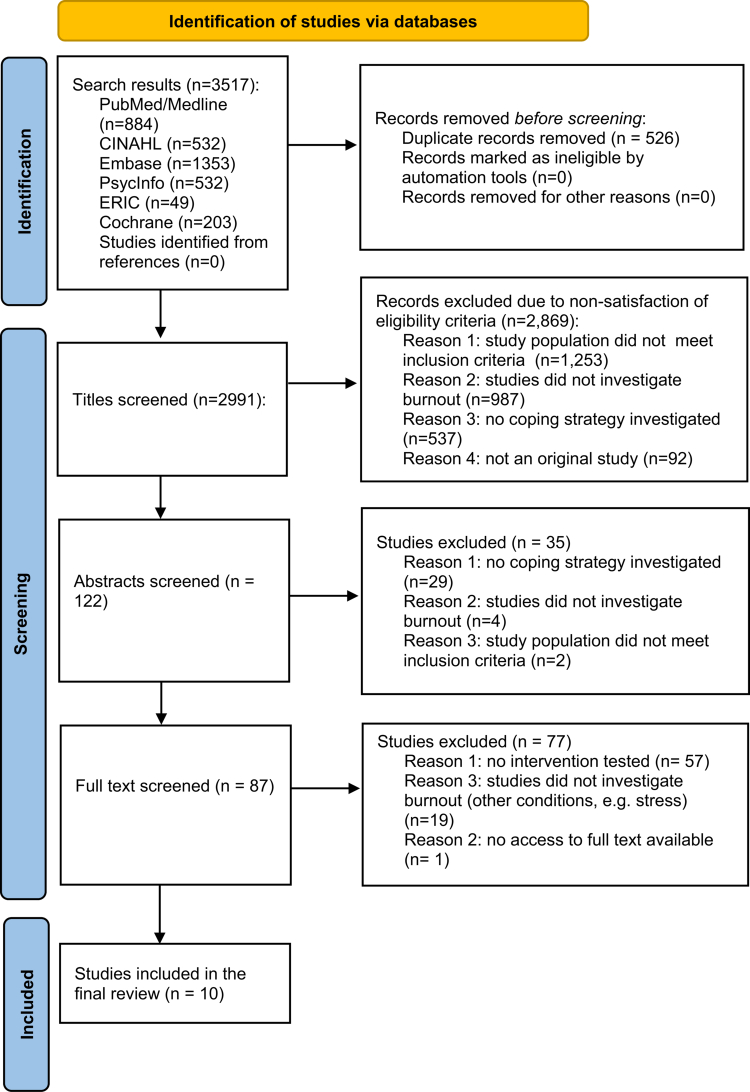
PRISMA flow chart of study selection process.

The search identified 3517 studies from six databases, of which 526 were removed for duplication, while the remaining were removed for not meeting the criteria, with the final selection of 10 studies meeting the inclusion criteria for this scoping review, as outlined in Figure 1 [28]. The finally selected publications' dates ranged between 2004 and 2023. Four of the studies were undertaken in the United States of America (USA), two in Spain, and one each in Australia, Chile, Indonesia, and Germany. A detailed summary of the included studies is presented in Table 1.

**Table 1.** Summary table of included studies.

Data charting						
Author, year	Title	Aim	Study population	Coping strategy/intervention tested	Method	Results
[29]-USA	Recreational music-making: an integrative group intervention for reducing burnout and improving mood states in first-year associate degree nursing students: insights and economic impact	To examine the impact of a Recreational Music-making (RMM) protocol on burnout and mood dimensions	79 nursing students	6-session Recreational Music-making (RMM)	Single Cohort study, compared pre- and post	At 6 weeks: Post intervention vs Pre intervention mean EE score ± SD (23.2 ± 11.8 vs 25.9 ± 11.7; *p* = 0.01). Post intervention vs Pre intervention DP (5.1 ± 5.3 vs 6.4 ± 6.6; *p* = 0.06). Post-intervention vs pre-intervention PA (34.3 ± 7.2 vs 34.4 ± 7.6; *p* = 0.35). Duration (at 12 weeks): EE 20.6 ± 11.4; *p* = 0.01 DP 6.0 ± 4.8; *p* = 0.45 PA 33.0 ± 9.2; *p* = 0.23
[30]-USA	A brief intervention to reduce burnout and improve sleep quality in medical students	To assess the impact of the sunrise alarm clock intervention on burnout scores in medical students	55 medical students	Sunrise alarm clock and electronic device abstinence	Single Cohort study, compared pre- and post	Post-intervention vs pre-intervention EE score (16.2 ± 9.2 vs 19.2 ± 8.3; *p* = 0.001). Post intervention vs pre-intervention DP score (9.9 ± 6.1 vs 12.5 ± 7.5; *p* = 0.001) Post-intervention vs pre-intervention PA score (36.7 ± 7.3 vs 34.9 ± 8.0; *p* = 0.023).
[31]-USA	Evaluating the effects of a mindfulness mobile application on student pharmacists' stress, burnout, and mindfulness	To evaluate the effectiveness of daily use of a mindfulness mobile application in improving student pharmacists’ burnout	56 pharmacy students	Mindfulness mobile application	Randomized controlled trial	EE 10.0 ± 8.8 in intervention group vs 20.0 ± 11.9 in the control group; *p* = 0.0061. DP 6.7 ± 5.8 in intervention group vs 14.3 ± 10.8 in the control group; *p* = 0.0086. PA 38.8 ± 6.8 in intervention group vs 30.6 ± 11.5 in the control group; *p* = 0.0139. Duration (at week 10): PA 39.0 ± 6.5 in the intervention group vs 30.4 ± 9.8 in the control group; *p* = 0.0101. EE and DP remained low at week 10 but were no longer significantly lower than in the control group.
[32]-Australia	Evaluation of an App-Delivered Psychological Flexibility Skill Training Intervention for Medical Student Burnout and Well-being: Randomized Controlled Trial	1)To evaluate the effectiveness of an app–delivered Acceptance and Commitment Training (ACT) intervention for improving medical students’ burnout. 2) To explore whether an individualized app would demonstrate benefits over a non-individualized version.	68 medical students	Psychological flexibility skill training	Randomized controlled trial	No statistically significant differences were found between the intervention arms and the control group. EE 14.48 ± 6.85 in individualized intervention group vs 14.79 ± 5.60 in non-individualized intervention group; *p* = 0.34 vs 17.05 ± 6.81 in control group; *p* = 0.75. DP 9.74 ± 5.70 in individualized intervention group vs 9.95 ± 5.54 in non-individualized intervention group; *p* = 0.44 vs 12.0 ± 7.62 in control group; *p* = 0.57. PA 27.5 ± 5.29 in individualized intervention group vs 26.4 ± 6.86 in non-individualized intervention group; *p* = 0.32 vs 23.9 ± 6.27 in control group; *p* = 0.05.
[33]-USA	Impact of a Mentorship Program on Medical Student Burnout	To evaluate the impact of a mentorship program on burnout in fourth‐year medical students during their 4‐week emergency medicine sub-internship	135 medical students and 59 medical residents participated as mentors.	Mentorship program	Two cohorts (intervention and control) were compared pre- and post	The difference between intervention and control groups was not statistically significant: EE *p* = 0.2, DP *p* = 0.5 PA *p* = 0.06 After potential confounders were adjusted for, there was a significant difference in PA scores (mean difference = 2.2 [0.1 to 4.3]; *p* = 0.04).
[34]-Indonesia	The Effect of Academic Self-Efficacy Training Toward The Nursing Students' Academic Burnout Participating In Block System Learning	To determine the effect of academic self-efficacy training on the academic burnout experienced by nursing students	90 nursing students	Academic self-efficacy training intervention	Randomized controlled trial	Intervention group vs control: EE 5.2 in intervention group vs 18.4 in control group. DP 10.7 in intervention group vs 15.1 in control group. Reversed PA 12.2 in intervention group vs 17.6 in control group. Independent Samples Test showed a significant difference favouring the experimental group in all domains *p* < 0.001. Intervention group pre- and post: EE 17.0 pre-intervention vs 5.2 post-intervention. DP 15.3 pre-intervention vs 10.7 post-intervention. Reversed PA 19.5 pre-intervention vs 12.2 post-intervention. The Paired Samples Test analysis showed *p* < 0.001, there was a significant difference in burnout pre- and post-intervention.
[35]-Spain	Lockdown, Emotional Intelligence, Academic Engagement and Burnout in Pharmacy Students during the Quarantine	To establish the effect of the current confinement on the teaching-learning process and academic performance, and the impact of the application of Emotional Intelligence on university students	47 second-year pharmacy students	Emotional intelligence training intervention	Single Cohort study, compared pre- and post	Post-intervention vs pre-intervention EE (4.31 ± 1.11 vs 5.26 ± 1.22; *p* < 0.01). Post-intervention vs pre-intervention DP (2.31 ± 1.39 vs 3.11 ± 1.08; *p* < 0.01). Post-intervention vs pre-intervention PA (4.63 ± 1.14 vs 3.15 ± 1.26; *p* < 0.001).
[36]-Spain	Effectiveness of a Mindfulness-Based Program on Perceived Stress, Psychopathological Symptomatology and Burnout in Medical Students	To evaluate and compare the effects of a mindfulness-based program on burnout in an experimental group compared to a control group.	143 medical students	Mindfulness training	Two cohorts (intervention and control) were compared pre- and post	No significant difference was found between groups in the level of burnout. EE 2.18 ± 0.93 in intervention group vs 2.28 ± 1.09 in control group; *p* = 0.743. DP 0.85 ± 0.81 in intervention group vs 0.95 ± 0.86 in control group; *p* = 0.218. PA 1.52 ± 0.71 in intervention group vs 1.94 ± 0.83 in control group; *p* = 0.884. Total MBI score 4.55 ± 1.89 in intervention group vs 5.18 ± 2.23 in control group; *p* = 0.677.
[37]-Germany	Strategies against Burnout and Anxiety in Medical Education—Implementation and Evaluation of a New Course on Relaxation Techniques (Relacs) for Medical Students	To examine the condition of students before and after the Relacs course.	127 medical students	Elective course for learning relaxation techniques: ‘Recreation and success in learning through applied concentrative self-relaxation (Relacs)’	Single Cohort study, compared pre- and post	The intervention group showed significant improvement in two of three aspects of decreased burnout. BOSS-II cognitive pre-intervention vs post-intervention (1.25 ± 0.87 vs 0.92 ± 0.72; *p* = 0.19). BOSS-II emotional pre-intervention vs post intervention (0.91 ± 0.79 vs 0.68 ± 0.64; *p* = 0.02) BOSS-II physical pre-intervention vs post intervention (1.27 ± 1.85 vs 0.83 ± 0.6; *p* = 0.2)
[38]-Chile	Reduced burnout and higher mindfulness in medical students after a self-care program during the COVID−19 pandemic.	To report the implementation and impact of an eight-week mindfulness program on medical students’ burnout during the COVID−19 pandemic	102 medical students	Mindfulness program	Single Cohort study, compared pre- and post	Proportion of students with burnout reduced significantly after intervention (48% pre vs 24% post; *p* = 0.002)

### Reviewed study design

Included studies were categorized based on study design according to the Research Guide by Georgia State University [39]. All of the reviewed studies were interventional, while five studies used a single-cohort design comparing pre- and post results [29,30,35,37,38]. Two studies compared an interventional cohort to a control group [33,36] and three studies were randomized controlled trials [31,32,34].

### Study populations

The sample size ranged between 39 and 143 participants. Six studies focused on the medical students [30,32,33,36–38]. Two studies focused on nursing students [29,34], and the remaining two studies focused on pharmacy students [31,35]. Table 2 lists study population characteristics at baseline.

**Table 2.** Characteristics of populations in included studies.

Author, year	Mean age (years)	Comorbidities	Study duration
**[29]**	27.5	Not reported	Two 6-week intervention periods (total duration 12 weeks)
**[30]**	24.8	Not reported	2 weeks
**[31]**	29.4	Not reported	6 weeks of intervention duration. Burnout was measured again at 10 weeks.
**[32]**	24	Not reported	5-week intervention duration
**[33]**	Not reported	Not reported	4 weeks
**[34]**	18	Not reported	2 weeks
**[35]**	20	Not reported	8 weeks
**[36]**	20.28	Not reported	16 weeks
**[37]**	24.29	Not reported	2012 summer term and 2012/2013 winter term.
**[38]**	Not reported	Not reported	8 weeks

### Study participant selection

Three studies recruited volunteers but incentivised participation with a monetary reward [30–32], while four studies with no reward [33,35–37]. For the remaining three, participation was mandatory [29,34,38].

### Burnout measurement

All of the reviewed studies used a validated tool to measure burnout, with the vast majority of studies using a version of the Maslach Burnout Inventory (MBI) survey. Four studies used a version of MBI adapted for the student population (MBI-GS) [32,34–36]. Two studies used MBI for human services workers (MBI-HSS) [30,38]. One study used MBI for medical personnel (MBI-HSS (MP)) [33]. Two studies used MBI survey not adapted to specific populations [29,31]. One study used the Burnout Symptom Scales (BOSS-II) [37].

### Burnout dimensions, interventions and efficacy

Coping strategies tested in three of the studies involved a form of mindfulness training [31,36,38], with conflicting results; two studies favored its effectiveness in reducing burnout in all three dimensions [31,38], while one concluded with no impact on burnout [36] Table 1.

Other coping strategies tested and found to be effective in reducing all three dimensions of burnout were emotional intelligence training [35], academic skills training [34], relaxation techniques training [37], and a combination of a morning alarm clock with abstinence from electronic devices at bedtime [30] Table 1.

Some coping strategies were found to be effective in improving some of the three dimensions of burnout. A recreational music-making program was effective in reducing two dimensions of burnout (EE and DP), but had no impact on the PA dimension [29]. A mentorship program intervention was effective in improving only the PA dimension of burnout, and had no impact EE or DP dimensions [33]. Psychological flexibility skill training intervention was found to be ineffective in reducing burnout [32].

Two studies also assessed the longevity of the effectiveness of interventions by measuring burnout after prolonged follow-up. Meditation using a mindfulness mobile application resulted in significant improvement in all burnout dimensions in the intervention group compared to the control group at 6 weeks; however, by 10 weeks, significance was only maintained in the PA dimension [31]. Likewise, recreational music initially significantly improved all burnout dimensions, but after 6 weeks, significance was again only maintained in PA dimension [29].

Overall, this scoping review identified 8 studies that contribute to the body of knowledge about interventions that successfully mitigate burnout in healthcare students.

#### Overall burnout dimensions improvement impact

Bittman et al reported that improvement in burnout dimensions was associated with an overall improvement in the Profile of Mood States (POMS) of nursing students [29]. POMS measures disturbances in one’s mood in terms of feelings of anxiety, hostility, dejection, vigor, fatigue, and confusion. Reduction in burnout was associated with improvement in all mood aspects aside from vigor [29]. Bittman et al also estimated that such results will prevent the dropout of 3−4 students per 100 nursing students [29].

#### Impact of electronic devices

Brubaker et al assessed the effect of a sunrise alarm clock and bedtime abstinence from electronics on sleep quality alongside its effect on burnout. Sleep quality was assessed using the Pittsburgh Sleep Quality Index (PSQI). At baseline, 77% of the study sample reported poor sleep quality, and at the end of the study, this reduced significantly to 43%, *p* = 0.026 [30]. This was associated with a significant increase in sleep duration from a mean of 6.7 hours to 7.1; *p* = 0.001 [30].

Chu et al assessed the effect of a mindfulness mobile application on mindfulness and stress, in addition to burnout [31]. Mindfulness was assessed using the Mindfulness Attention Awareness Scale (MAAS), while stress was assessed using the Perceived Stress Scale (PSS). As expected, the mindfulness application improved (increased) mindfulness scores in the intervention group significantly at 10 weeks follow-up compared with the control group, with a mean score ± SD of (4.4 ± 0.7 versus 3.4 ± 0.9); *p* < 0.001 [31]. Similarly, perceived stress levels were significantly better (lower) in the intervention group compared with the control group mean ± SD of (14.6 ± 5.7 versus 23.2 ± 7.70; *p* < 0.001 [31].

#### Well-being and psychological impact

Ditton et al looked at a range of secondary outcomes, including well-being and psychological distress [32]. Wellbeing was measured using the Mental Health Continuum Short Form (MHC-SF), whereas Distress was measured using the Depression Anxiety and Stress scale−21 (DASS−21), both tools being validated for their respective outcomes [32]. Those receiving the intervention reported significantly better (higher) mean ± SD wellbeing scores compared with the control group (52.1 ± 9 versus 40.4 ± 13); *p* = 0.040. Mean ± SD Perceived depression significantly improved (reduced) in the intervention group compared with the control group (7.7 ± 7 vs 11.7 ± 9); *p* = 0.046. Similarly, mean ± SD perceived stress was also significantly better (lower) in the intervention group compared with control (10.0 ± 7 vs 15.8 ± 9); *p* = 0.030 [32]. There was, however, no significant difference in perceived anxiety between the intervention and control group, with a mean ± SD of (6.4 ± 7 versus 7.1 ± 6); *p* = 0.170 [32].

#### Impact of mentorship programs

Jordan et al assessed the impact of a mentorship program on stress, career guidance and professional development. Mentorship was associated with a reduction in stress levels in 28/48 (58%) of participants, 35/48 (73%) reported that mentorship provided strong career guidance, and 39/48 (81%) felt strong professional development as a result of mentorship [33].

#### Mindfulness and emotional wellbeing

Moreno-Fernandez et al assessed engagement with studies using the Utrecht Work Engagement Scale for students (UWES-S), this tool has three dimensions which are Vigor, Dedication and Absorption. All three dimensions saw significant improvement associated with emotional intelligence training. Mean ± SD Vigor improved (increased) significantly after intervention, (3.8 ± 1 versus 2.5 ± 1); *p* < 0.010 [35]. Similarly, Dedication significantly improved (increased) after intervention, (5.2 ± 1 versus 3.4 ± 1); *p* < 0.010. Likewise, Absorption also significantly improved (increased), (4.2 ± 1 versus 3.1 ± 1); *p* < 0.010 [35].

Oró et al. measured psychological distress using the Symptom Checklist -90- Revised (SCL−90-R) and perceived stress using the PSS [36]. The SCL−90-R results at baseline showed that medical students' levels of psychological distress were higher (worse) than those of the reference values for the general population [36]. After mindfulness training, there was a significant improvement compared with baseline in SCL−90-R scores in the dimensions of somatisation, obsessive compulsiveness, interpersonal sensitivity and anxiety; *p* = 0.014; 0.022; 0.005; 0.014, respectively [36]. PSS results showed that mindfulness training reduced perceived stress significantly in the intervention group compared to the control group, mean ± SD of (20.1 ± 5 versus 22.8 ± 7); *p* = 0.005 [36].

Wild et al assessed the use of relaxation techniques on anxiety levels using the State and Trait Anxiety Inventory (STAI-G) [37]. Mean ± SD value for Anxiety trait reduced significantly with post-intervention compared with baseline (42.3 ± 5 versus 45.1 ± 7); *p* = 0.010 [37].

Beck’s Depression Inventory II was used to assess depressive symptoms, although no statistical hypothesis testing was reported for this outcome, the authors reported that there was a reduction (improvement) in depressive symptoms associated with relaxation techniques training [37]. Coherence was also measured pre- and post-intervention using the Sense of Coherence Scale (SOC-L9), again, there was no statistical hypothesis testing of this outcome, but the authors reported increased (improved) coherence [37].

Zúñiga et al measured the effect of a mindfulness training program on perceived stress using the PSS, coping strategies using the Brief Coping Orientation to Problems Experienced Questionnaire (Brief-COPE), and Resilience using the Connor-Davidson Resilience Scale (CD-RISC) [38]. The success of acquiring a mindful disposition was assessed using the Mindfulness Attention Awareness Scale (MAAS) [38]. After the intervention, the proportion of students with a high mindfulness disposition on MAAS increased significantly from 25% to 44%; *p* = 0.002 [38]. This was associated with a significant drop (improvement) in mean perceived stress on PSS compared with baseline (15.1 versus 19.9); *p* < 0.001. It was also associated with higher (improved) Brief-COPE scores after intervention compared with baseline, mean Active coping of (13.3 versus 12.4); *p* < 0.001, support seeking of (11.7 versus 10.9); *p* = 0.004, and positive reframing of (6.0 versus 5.6); *p* = 0.003, all of which implies that students were adopting healthier coping strategies [38]. Mean CD-RISC also increased (improved) significantly compared with baseline (29.8 versus 27.7); *p* < 0.001 [38].

## Discussion

This scoping review summarizes current knowledge on effective interventions for treating burnout among healthcare students. Training in mindfulness, academic skills, emotional intelligence and relaxation techniques were all identified to improve all dimensions of burnout [31,34,35,37,38]. Abstinence from electronics at bedtime, combined with a sunrise morning alarm clock, was also found to be effective in improving all dimensions of burnout [30]. The longevity of the effectiveness of these interventions lasted up to twelve weeks [29,31]. Although twelve weeks is not a long period, a simple intervention such as abstinence from electronics combined with a sunrise alarm clock can be implemented yearlong or even lifelong, rather than for a brief experimental period. Likewise, a mobile phone application for relaxation and meditation should be accessible long-term. Furthermore, when considering that burnout is a maladaptive response to occupational stresses, these interventions may be instituted at the beginning of higher education to instil a healthy response and prevent maladaptive ones from being established.

Within Kohn’s proposed categories for coping mechanisms, eight studies were emotion oriented [29,31,32,34–38] and two were problem oriented [30,33]. Three of the studies used in-person teaching for their interventions [29,36,37], one study used online teaching [35], and a further three studies utilized a mixture of both [33,34,38]. Two studies used a mobile application to deliver their interventions [31,32], and one study used an alarm clock [30]. These variations highlight two important issues in the literature on burnout; firstly, there is a wide breadth of available interventions that universities can deploy to aid their students in tackling burnout. Secondly, the current heterogeneity of approaches indicates that research on burnout in healthcare students is at an early phase, where potentially effective interventions are still being explored without an established hierarchy of primary interventions. However, in due course, focus may shift towards optimizing those that are proven to be effective and cost-effective interventions to create this hierarchy.

Of note, one study on a mentorship program found that it only improved the PA dimension of burnout [33]. This result comes as a surprise to established wisdom, as additional professional support should correlate positively with reduced stress and, by extension, burnout [40,41]. The design of intervention in Jordan et al’s study may have played a role in this result, however. The authors reported difficulties in maintaining the mentorship relationship, as 37% of mentor-mentee pairs failed to meet the minimum required times. This reduced compliance with the intervention activities is likely to have influenced study results. However, this limitation and by extension the wider study findings may reflect real-world findings as pressures on healthcare providers have increased globally, and perhaps adding further mentorship responsibility may not be deliverable. Perhaps student-guided mentorship programs may offer a more readily deliverable source of support and promote positive strategies for coping with burnout [42], as senior healthcare students can more easily relate to challenges encountered by junior students in the same field [43]. Many studies have demonstrated that such programs help in preventing burnout and easing anxiety and stress, known precursors as well as consequences of burnout [40,44,45].

As previously mentioned, burnout is a distinct entity but is closely related to depression and stress, and all three can overlap and coexist. It therefore follows that interventions to relieve burnout can also relieve a wider range of conditions and maladaptive behaviors. Analogously, some of the maladaptive behaviors and underlying causes that lead to burnout can also lead to these conditions. It is therefore welcome that many of the studies in this review also demonstrated wider benefits to interventions on burnout, which included improved mood state, sleep quality, reduced perceived stress, improved wellbeing and an increase in resilience [29,31,32,38]. Further, one study showed that universities should be incentivised to invest in these interventions as they result in better retention of students and are ultimately cost effective as the retention of students increases income; furthermore, student retention should result in additional graduates entering the workforce, which would provide much needed boost to all healthcare services [29].

Even where interventions did not significantly improve burnout, other benefits were reported, as was the case in the mentoring scheme study [33]. Mentees reported lower stress levels with studies, improved professional development and clearer direction towards career goals [33]. These positive side effects demonstrate the value of investment in research and that negative results should not discourage researchers or funders from investing in these studies. Many of the studies incorporated their interventions as part of university curricula, demonstrating that tackling burnout can be streamlined alongside delivering necessary teaching [29,31,33,35].

### Limitations and strengths

While this scoping review offers valuable insights into the current state of research on academic burnout among healthcare students, there are certain limitations which can be categorized into issues inherent in scoping reviews and those specific to the included studies. Some key limitations are the exclusion of non-English language studies, potentially missing relevant findings from other linguistic contexts, as well as the reliance on certain databases, which may have restricted the scope of the literature search. However, the oversight by a university-appointed Project Supervisor adds a layer of assurance regarding study meticulousness.

The impact of the COVID−19 pandemic on healthcare education is acknowledged, with a shift towards reduced clinical contact, more distance learning, and limited networking opportunities. The temporal limitations of studies conducted before and during the pandemic are recognized, influencing the generalizability of their findings to the current student population. Nevertheless, the studies included in the review provide a foundation for raising awareness and implementing measures against burnout.

Methodological limitations of scoping reviews, such as the lack of critical appraisal of included studies, are acknowledged. The relatively modest sample size of around 900 participants and the exclusion of certain allied health professions may limit its implications. The use of monetary rewards or mandatory participation in some studies may lead to reporting bias in self-reported burnout measurements. Notably, none of the included studies addressed important confounding factors such as physical or mental health, socioeconomic status, family support, hobbies, or part-time employment. Despite these limitations, the review has several strengths. The adoption of Arksey and O’Malley’s established methodological framework for scoping reviews provides a structured and systematic approach. The oversight by an educational supervisor ensures methodological accuracy and rigor. Collaboration with an academic librarian for search strategy design enhances the likelihood of identifying relevant literature. The inclusion of mostly recent studies within the last decade contributes to the review's up-to-date nature. Identified gaps in research highlight the need for re-evaluating interventions in the post-pandemic education landscape and the necessity of inclusive studies across healthcare disciplines. The absence of research on protective factors against burnout emphasizes the importance of exploring preventive strategies. Future studies are encouraged to address confounders, incorporate diverse outcome measures, and consider longitudinal approaches.

## Conclusion

In conclusion, this scoping review consolidates evidence on effective treatments for burnout in healthcare students. Mindfulness, emotional intelligence, relaxation techniques, and certain lifestyle modifications show promise in reducing burnout. The positive correlative effects of interventions, even when burnout reduction is not significant, underscore the broader benefits of addressing student well-being. The review advocates for continued research, emphasizing the importance of longitudinal studies to assess the long-term impact of burnout interventions on student outcomes and urging a more nuanced understanding of intervention effectiveness based on individual variations and thresholds [46–51].

## Supplementary Material

Supplementary material
