# Supplementary material for: Academic burnout and coping strategies in healthcare students: a scoping review
Source: Med Educ Online. 2025 Nov 9;30(1):2579392. doi: 10.1080/10872981.2025.2579392 (PMC12604133; doi:10.1080/10872981.2025.2579392)
Supplement: Supplementary material [file ZMEO_A_2579392_SM1030.docx]

**Search Strategy**

This scoping review was conducted in accordance with the methodological framework developed by Arksey and O’Malley (2005) and later enhanced by Levac et al. (2010). The framework comprises the following stages: identifying the research question, identifying relevant studies, study selection, charting the data, and collating, summarising, and reporting the results.

**Information Sources and Search Terms**

A comprehensive literature search was conducted on March 6, 2023, with support from a subject librarian. The following electronic databases were searched:

- Medline/PubMed
- CINAHL
- Embase
- PsycINFO
- ERIC
- Cochrane Library

Search terms included both free-text keywords and MeSH terms. The core search terms included:

- Burnout
- Coping OR Cope OR Help OR Manage
- Student OR Academic

Search syntax followed Boolean logic. For example: burnout AND (coping OR cope OR help OR manage) AND (student OR academic). Where available, MeSH subject headings were employed to broaden the search by capturing synonymous terms (e.g., “student,” “pupil,” “trainee”).

**Search Strategy Example**

Database search was structured as follows:

1. Search all titles and abstracts containing “burnout”.
2. Perform a MeSH search for the term “student” to capture synonymous terms.
3. Conduct a MeSH search for “coping”.
4. Combine the above results to produce final eligible hits.

**Study Selection**

The study selection process included three stages:

1. Deduplication of search results
2. Title and abstract screening to exclude irrelevant studies
3. Full-text screening of the remaining articles against the eligibility criteria

Additionally, reference lists of included studies were screened to identify any potentially relevant articles missed in the initial search.
